# Supplementary material for: The Intrinsically Disordered N Terminus in Atg12 from Yeast Is Necessary for the Functional Structure of the Protein
Source: Int J Mol Sci. 2023 Oct 10;24(20):15036. doi: 10.3390/ijms242015036 (PMC10606595; doi:10.3390/ijms242015036)
Supplement: Supplementary file 1 [file ijms-24-15036-s001.zip › ijms-2587518-supplementary.pdf]

SUPPLEMENTARY MATERIAL

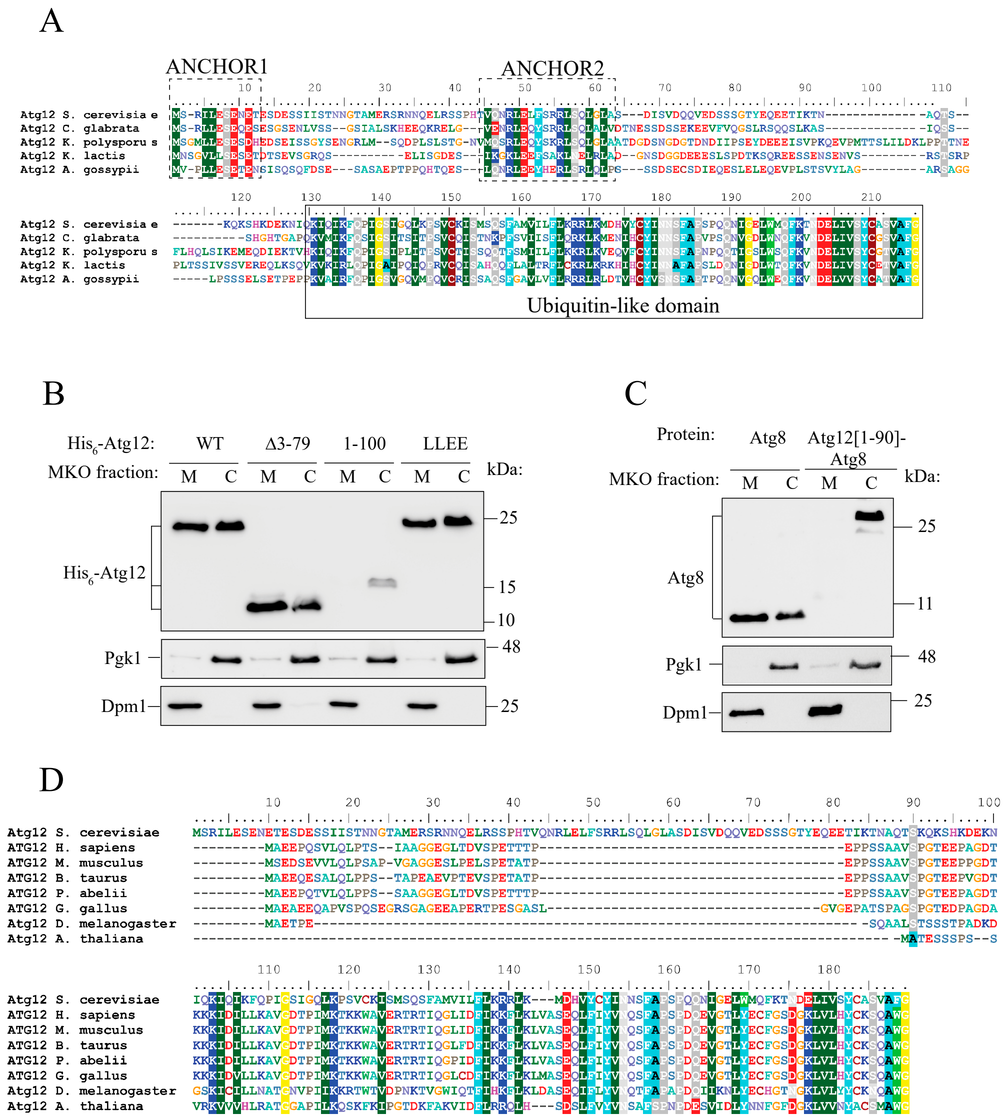

Supplementary Figure S1. The IDPR of Atg12 from fungi has two homologous binding elements that do not associate the protein with cellular membranes. **A** Multiple amino acid sequence alignments for the Atg12 protein from various fungi. The UBL domain is highly conserved, whereas the N-terminal IDPR only exhibits two highly conserved segments. These segments overlap with the predicted ANCHOR regions, which cannot fold on their own but gain, based on their physiochemical properties, stabilizing energy via binding to a globular binding partner. **B** Subcellular fractionation of multiple-knockout (MKO) cells expressing His$_6$-Atg12 variants; LLEE is L54E L57E. Pgk1 and Dpm1 serve as controls for the cytosolic (C) and membrane (M) fraction, respectively. **C** Subcellular fractionation of MKO cells expressing Atg8 and the Atg12[1-90]Atg8 chimeric protein. **D** Multiple amino acid sequence alignments for the Atg12 protein from various organisms, including flies, plants, birds, and mammals.

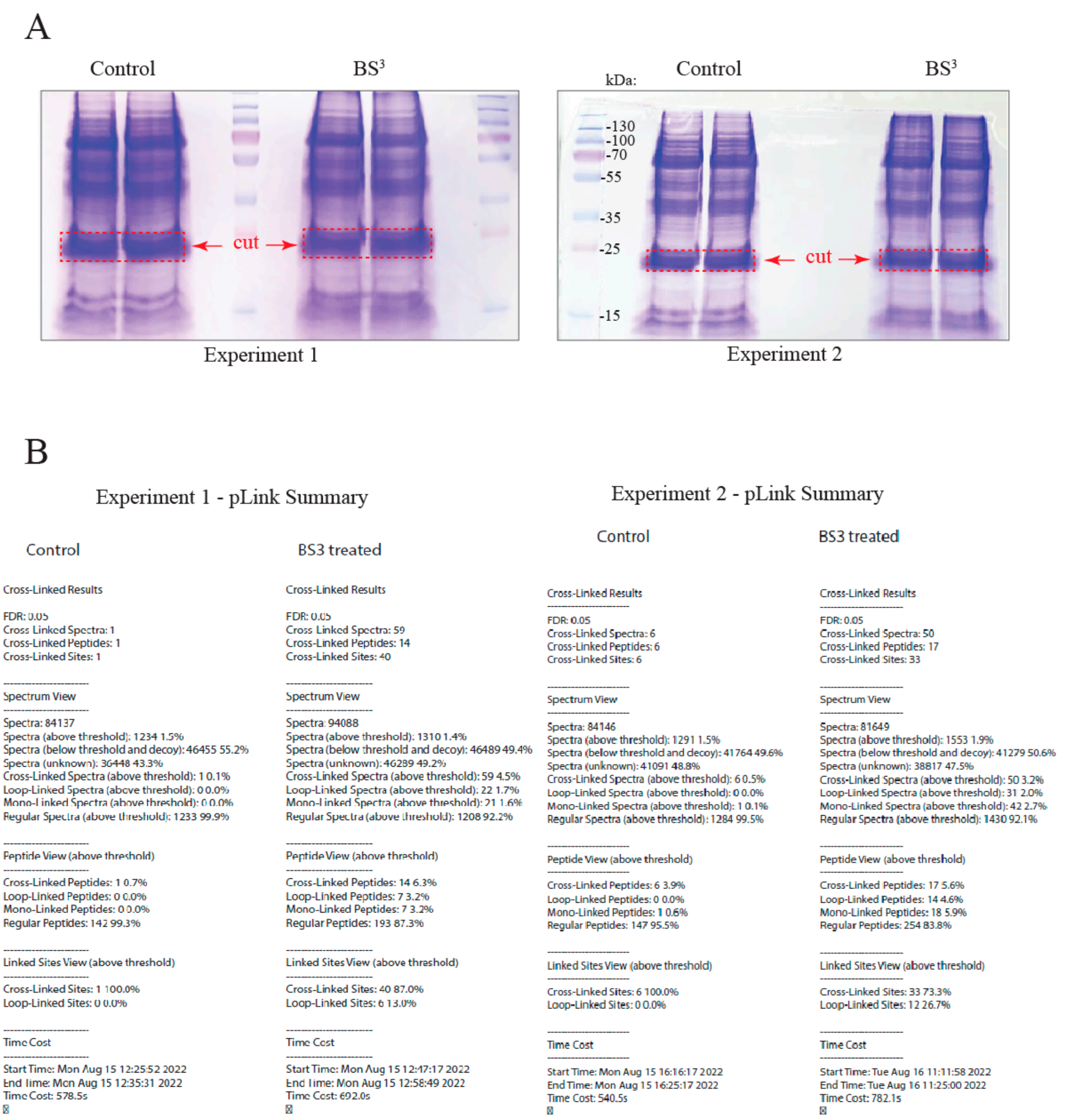

Supplementary Figure S2. Crosslinking mass spectrometry. **A** Coomassie Brilliant Blue-stained SDS-PAGE gels of recombinant, partially purified His_6-Atg12 that was expressed in *E. coli* cells along with Atg7 and Atg10. The protein band (red rectangle) corresponding to full-length unconjugated Atg12 (22 kDa) with or without BS^3 crosslinking was cut from the gel and subjected to XL-MS analysis. **B** Summary of the results from the pLink algorithm that evaluated the raw XL-MS data.

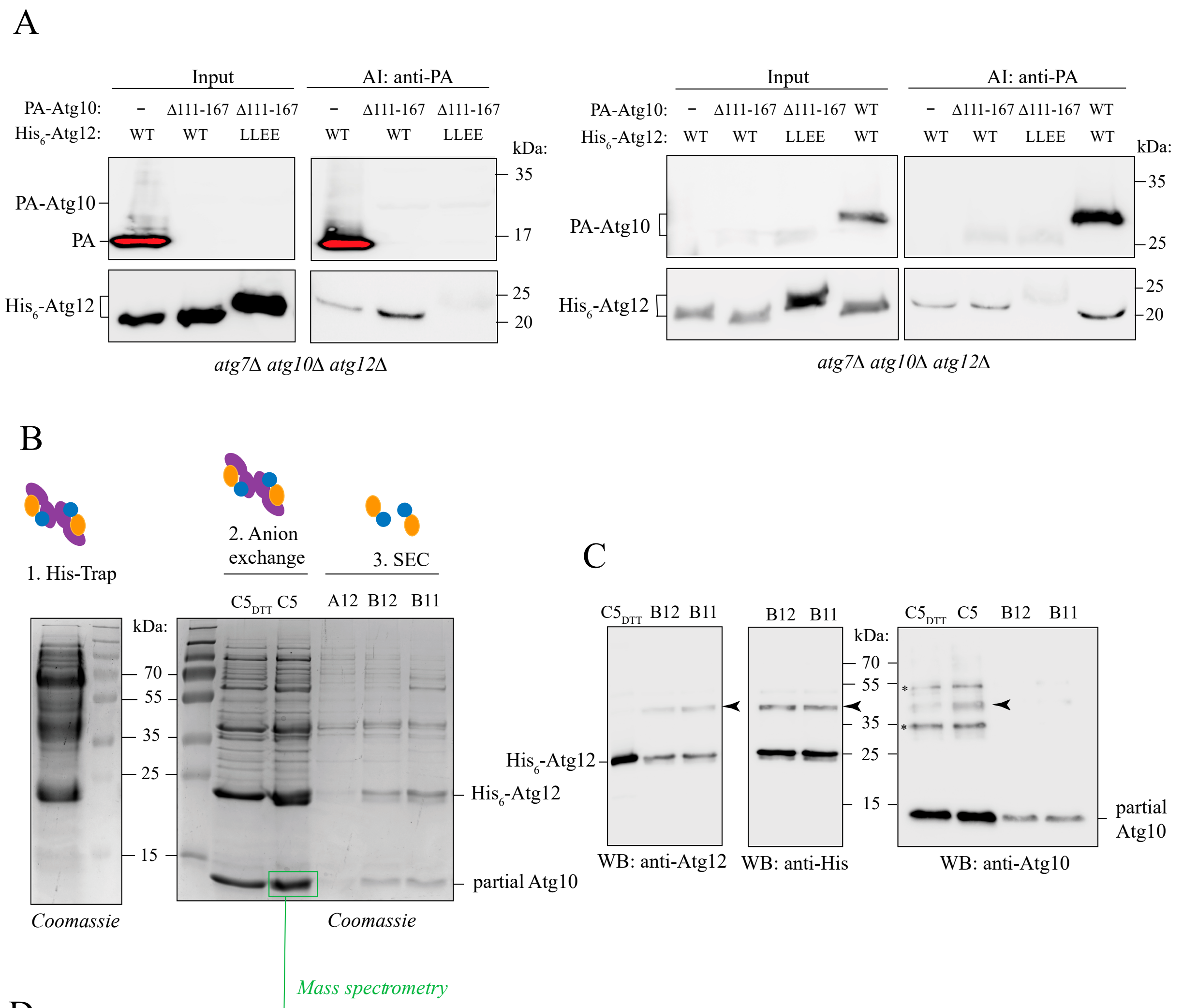

D

Accession #	*Description*	*Covarage (%)*	*Peptides*	*#PMS*	*Unique peptides*	*#AA*	*MW(kDa)*	*Calc. pI*
Q07879	Ubiquitin-like-conjugating enzyme ATG10 OS=Saccharomyces cerevisiae (strain ATCC 204508 / S288c) OX=559292 GN=ATG10 PE=1 SV=1	50	5	14	5	167	19.7	4.94

# Proteins	# PSMs	Master Protein Accessions	Positions in Master Proteins	Modifications	# Missed Cleavages	Theo. MH+ [Da]
1	1	Q07879	Q07879 [1-35]		0	4374.98592
1	1	Q07879	Q07879 [1-35]		0	4389.0128
1	2	Q07879	Q07879 [72-81]		0	1229.72523
1	1	Q07879	Q07879 [87-95]		0	977.49721
1	3	Q07879	Q07879 [59-71]		0	1606.88392
1	2	Q07879	Q07879 [59-71]		0	1607.86794
1	2	Q07879	Q07879 [96-111]		0	1771.95101
1	2	Q07879	Q07879 [96-111]		0	1787.94593

Found in Sample:	Confidence (by Search Engine): A2 Sequest HT	XCorr (by Search Engine): A2 Sequest HT	Sequence in Protein	Positions in Proteins
High	High	6.53	-.MIPYQEWHSQLQSLYDSQIFHNWALCQDVHLNDEK.D	[1-35]
High	High	5.57	-.MIPYQEWHSQLQSLYDSQIFHNWALCQDVHLNDEK.D	[1-35]
High	High	3	K.VYNEPLLLLR.I	[72-81]
High	High	1.9	K.SIDGIPMTK.L	[87-95]
High	High	4.26	K.LLNHIELYLTYSK.V	[59-71]
High	High	2.87	K.LLNHIELYLTYSK.V	[59-71]
High	High	5.2	K.LMLPTDIESLLDVQGK.F	[96-111]
High	High	4.41	K.LMLPTDIESLLDVQGK.F	[96-111]

Supplementary Figure S3. Atg12 binds partial Atg10. **A** Two independent affinity-isolation experiments with *atg7*Δ *atg10*Δ *atg12*Δ yeast cells overexpressing His_6-Atg12 wild-type protein or the L54E L57E (LLEE) mutant along with free PA, PA-Atg10, or PA-Atg10[Δ111-167] under the control of the *CUP1* promoter. To better detect PA-Atg10[Δ111-167] (affinity isolation on the right), free PA was cut off before incubation of the PVDF membrane with the anti-PAP antibody **B** Purification of recombinant proteins from *E. coli*. Coomassie Brilliant Blue-stained SDS-PAGE gels show His_6-Atg12-containing fractions after purification of bacterial cell lysates by gravity-flow chromatography on Ni-NTA agarose followed by anion exchange chromatography on a HiTrap Q HP column and then size-exclusion chromatography (SEC) on a Superdex 75 column. Recombinant proteins expected to be in the purification fractions are schematically depicted in different colors: Atg7, purple; Atg10, orange; Atg12, blue. **C** Western blot analysis of the C5 fraction from the anion exchange chromatography and B12 and B11 fractions from the SEC. $C5_{DTT}$ is the C5 fraction supplemented with 100 mM DTT. Black arrows mark the Atg10–Atg12 thioester conjugate of 42 kDa (Atg10, 20 kDa; Atg12, 22 kDa) that is sensitive to DTT. Asterisks denote nonspecific bands. **D** Mass spectrometry analysis of the 13 kDa protein band (green rectangle in B) excised from the Coomassie Brilliant Blue-stained gel.

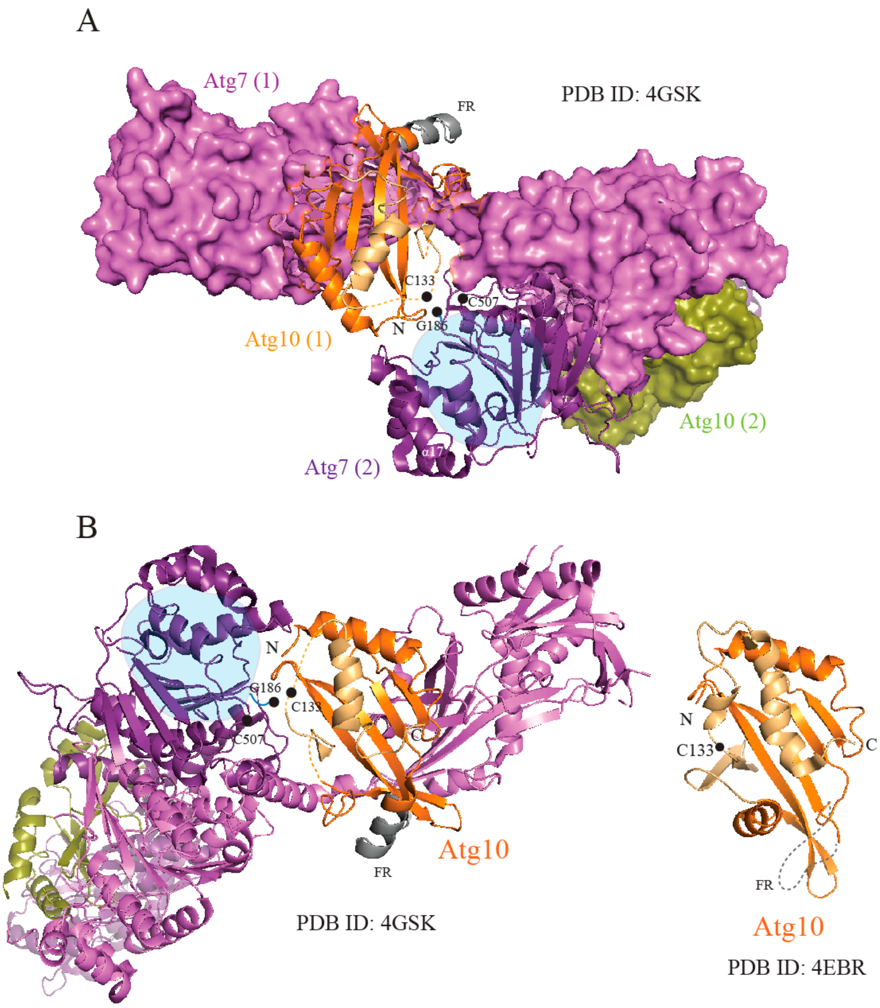

Supplementary Figure S4. Crystal structures of Atg7 and Atg10. **A** Crystal structure of the Atg7–Atg10 heterotetramer (PDB ID: 4GSK). The space where the Atg12 UBL was modeled (Kaiser et al. 2012) is depicted by a light blue cloud. FR denotes the α helix (gray) that is folded in Atg10 from a flexible region (FR). **B** Crystal structure of the Atg7–Atg10 heterotetramer (PDB ID: 4GSK), shown to visualize Atg10 in the same orientation as the protein in the crystal structure of individual Atg10 (PDB ID: 4EBR). Note that the FR of Atg10 crystalized in the absence of Atg7 (PDB ID: 4EBR) is a disordered loop (gray dashed line), whereas the same region forms an α helix (gray) in the Atg7–Atg10 heterotetramer.

Table S1. *S. cerevisiae* strains used in this study.

Name	Genotype	Reference
HPY040	*SEY6210 atg12Δ::HIS*	This study
HPY043	*SEY6210 atg12Δ::KAN atg7Δ::HIS*	This study
HPY044	*SEY6210 atg12Δ::KAN atg7Δ::HIS atg10Δ::LEU*	This study
HPY087	*SEY6210 vac8Δ::KAN atg12Δ::HIS*	This study
SEY6210	*MATα leu2-3,112 ura3-52 his3-Δ200 trp1-Δ901 lys2-801 suc2-Δ9 GAL*	Robinson et al. 1988
YCY131 (MKO)	*SEY6210 atg1Δ, 2Δ, 3Δ, 4Δ, 5Δ, 6Δ, 7Δ, 8Δ, 9Δ, 10Δ, 11Δ, 12Δ, 13Δ, 14Δ, 16Δ, 17Δ, 18Δ, 19Δ, 20Δ, 21Δ, 23Δ, 24Δ, 27Δ, 29Δ, 31Δ::ble*	Cao et al. 2008

Supplementary References:

Kaiser S.E., Mao K., Taherbhoy A.M., et al, 2012. Noncanonical E2 recruitment by the autophagy E1 revealed by Atg7-Atg3 and Atg7-Atg10 structures. Nat Struct Mol Biol. 19(12), 1242-1249.

Cao, Y., Cheong, H.S., Song, H., and Klionsky, D.J.,2008. In vivo reconstitution of autophagy in Saccharomyces cerevisiae. J Cell Biol 182, 703-713.

Robinson, J.S., Klionsky, D.J., Banta, L.M., and Emr, S.D.,1988. Protein Sorting in Saccharomyces-Cerevisiae - Isolation of Mutants Defective in the Delivery and Processing of Multiple Vacuolar Hydrolases. Mol Cell Biol 8, 4936-4948.
